# Investigation of the Relationship of Impacted Maxillary Canines with Orthodontic Malocclusion: A Retrospective Study

**DOI:** 10.3390/children10060950

**Published:** 2023-05-26

**Authors:** Orhan Cicek, Turhan Gurel, Busra Demir Cicek

**Affiliations:** 1 Department of Orthodontics, Faculty of Dentistry, Zonguldak Bulent Ecevit University, Zonguldak 67600, Türkiye; 2 Zonguldak Provincial Health Directorate, Zonguldak Oral and Dental Health Center, Zonguldak 67020, Türkiye

**Keywords:** impacted maxillary canine, malocclusion, relationship, orthodontics

## Abstract

Impacted canines, which play an important role in smile aesthetics and functional occlusion, can lead to dental and skeletal malocclusions. In this study the aim was to evaluate the relationship between impacted maxillary canines and malocclusion. A total of 151 patients comprising 101 females and 50 males aged between 13 and 33 years were included. The groups were divided based on age, gender, skeletal and dental classification, and sector classification. Angular and linear measurements were performed on lateral cephalometric and panoramic radiographs. In panoramic radiographs, the vertical distance of the impacted canine to the occlusal plane and the angle between it and the bicondylar plane were measured and sector classification was performed according to its relationship with the root of the lateral incisor. Skeletal classification was performed according to the ANB angle on lateral cephalometric radiographs and dental classification by molar relationship via the intraoral photographs. The Chi-square test analyzed independent qualitative and quantitative data using Kruskal–Wallis and Man–Whitney U tests. The statistical significance level was accepted as *p* < 0.05. According to the intraclass correlation test, an excellent positive correlation was found with 0.985 for canine distance and 0.993 for canine angle between the repeated measurements. The impaction of the maxillary right canine was significantly highest in females and lowest in males. The impacted canine angle was significantly highest in sector 1 and lowest in sector 4. Distance to the occlusal plane was significantly higher in dental Class II and sector 4. It was observed that there was a considerable relationship between impacted maxillary canines and malocclusion; bilateral impacted canines were more frequent in skeletal Class III, and the distance of impacted canines to the occlusal plane increased while their angles decreased both in dental Class II and from sectors 1 to 4.

## 1. Introduction

Orthodontic treatment should align the teeth in accordance with the ideal occlusion, even though it is not always possible to achieve goals such as improving chewing functions and enhancing facial and smile aesthetics [1]. Considering their position in the mouth, impaction of the maxillary canines, which support both the alar base and the upper lip, poses a significant challenge to achieve these goals [1,2].

Crown calcifications of the maxillary canines, which begin to develop in the fourth and fifth months, are completed at the age of six to seven years and erupt between the ages of eleven and twelve during the normal development period [3,4]. However, since the maxillary canines, which have an important role in managing the growth of the jaw in the correct direction, have a long and complex eruption path under the guidance of the lateral teeth, any deviation that may occur in this path may result in impaction [5].

Impaction occurs when a tooth is prevented from erupting in the mouth normally due to bone, soft tissue, or neighboring teeth [6]. This can be confirmed through clinical examination and radiography [3]. A tooth is considered as impacted if it has not erupted after its root has fully developed, or if the tooth in the opposing arch has erupted for more than six months and has a fully developed root [7].

Maxillary canines are the most commonly impacted teeth after the third molars and are considered to affect 2% of the population. The incidence of impacted teeth is two times higher in the maxilla than in the mandible and is more common in younger ages and females. Bilateral impacted canines are observed in approximately 8% of affected patients. The main causes of impacted maxillary canines may be genetic, localized, or systemic [8]. Local etiologic factors include the ectopic position of the tooth germ, long tooth root, lack of lateral incisors, lack of space for tooth eruption, and crowding [6]. For these reasons, maxillary canines may remain impacted in the palatal or labial position. The incidence of palatally impacted teeth is two times higher than the incidence of labially impacted teeth [8].

Impacted maxillary canines may cause different complications such as external root resorption and loss of vitality in adjacent teeth, shortening of the existing arch length, causing follicular cyst, development of ankylosis, infection focus, or pain. Comprehensive clinical examination and diagnosis aims to prevent complications and damage to surrounding tissues and teeth and malocclusions that may occur [9].

Orthodontic malocclusion is defined as any deviation from the normal relationship of the teeth in the same arch with each other and with the teeth in the opposite arch [10]. In maxillary canine impaction, which is a malocclusion that is difficult to treat, the horizontal position, vertical height, and bucco-palatal position of the impacted canine affect the prognosis [11]. Pop et al. [7] reported that impacted canines were most commonly associated with Class II Division 2 malocclusion. In addition, a distance between the impacted canine and the occlusal plane of more than 14 mm, increased impacted canine angle, and bilateral impaction increase the duration of treatment [12,13,14].

Various radiographic evaluation tools have been used for the evaluation and classification of impacted maxillary canines, and detailed examination of the position, angle, and orientation of impacted canines is essential for orthodontic treatment [15]. Although there are previous studies investigating the relationship and prevalence of impacted canines with various malocclusions [11,16,17], to the best of our knowledge, there is no comprehensive study investigating the distance to the occlusal plane and the angle with respect to the bicondylar plane of impacted maxillary canines according to gender, age, sector classification, dental classification, and skeletal classification. Therefore, in this study, our aim was to investigate the relationship of impacted maxillary canines with malocclusion comprehensively, thus providing an important diagnosis in the orthodontic treatment prognosis.

The first null hypothesis of the study was that there is no difference in age, gender, dental classification, skeletal classification, and sector classification in terms of the angle between the impacted maxillary canines and the bicondylar plane. The second null hypothesis of the study was that there is no difference in age, gender, dental classification, skeletal classification, and sector classification in terms of the distance of the impacted maxillary canines to the occlusal plane.

## 2. Materials and Methods

The material of this retrospective study consisted of a total of 151 patients, 101 females (mean age: 17.06 ± 3.9) and 50 males (mean age: 15.5 ± 2.6), ranging in age from 13 to 33 years (mean age: 16.56 ± 3.6), who applied to the Zonguldak Bülent Ecevit University Department of Orthodontics with the complaint of unilateral or bilateral impacted maxillary canines. This study was designed retrospectively by examining lateral cephalometric radiographs, panoramic radiographs, and intraoral photographs over clinical archive records. Ethical approval for the study was obtained from the Zonguldak Bülent Ecevit University Non-Interventional Clinical Research Ethics Committee dated 23 November 2022, with decision number 2022/20-2.

For diagnosis, linear and angular measurements were performed on panoramic radiographs taken with a panoramic X-ray machine (Veraviewepocs 2D, J Morita Mfg. Corp., Kyoto, Japan) and lateral cephalometric radiographs taken with a cephalometric X-ray machine (Veraviewepocs 2D, J Morita Mfg. Corp., Kyoto, Japan). The positions of the impacted maxillary canines were determined by examining the cone-beam computed tomography (CBCT) images taken on a tomography machine (Veraviewepocs 3D R100 /F40, J Morita Mfg. Corp., Kyoto Japan). Lateral cephalometric radiographs were taken at rest and in the natural head position. When taking panoramic radiographs, it was ensured that the Frankfurt horizontal plane was parallel to the ground and the bite bar was bitten in the correct position to ensure standardization. The inclusion criteria for the study were as follows: history of impacted maxillary canine, age 13 years and older, no previous orthodontic treatment, no history of trauma, no congenital anomalies, high-quality intraoral photographs taken at the accurate angle, and panoramic and lateral cephalometric radiographs with high resolution and good image quality. If at least one of the inclusion criteria was not met, the patient was excluded from the study.

The sample size of the study, in which the effect size was calculated as 0.52 using the group mean and standard deviation, was performed with the G*Power program (version 3.1.9.7; Franz Faul, Universität Kiel, Kiel, Germany). When the α error probability was set to 0.05 and the power (1 − β error prob) of the study was to be 0.90, according to these data, the actual power of the study was calculated as 90%, and the total sample size should have been 126. A total of 151 samples meeting the inclusion criteria were included in the study, from patients who applied to the clinic between 2014 and 2022, as in the previous study of Ajami et al. [11]. Since this study was retrospective and consent was obtained from all patients before orthodontic treatment that the archive records could be examined for use in scientific studies, no additional consent was obtained.

Impacted maxillary canines were evaluated separately as right impacted (tooth number 13), left impacted (tooth number 23), and bilaterally impacted (tooth numbers 13 and 23). In the study, groups were formed according to age, gender, angle classification, skeletal classification, and sector classification. Based on age, the groups were divided into 13–16 years, 17–20 years, and 21 years and older. The data of the patients are presented in Table 1.

The sagittal skeletal directional relationship of the jaws was evaluated according to Steiner’s ANB angle measured using the NemoCeph (Nemotec, 2006, Madrid, Spain) digital analysis program on lateral cephalometric radiographs. The ANB angle is the angle between the hard tissue A point–Nasion-B point [18]. Accordingly, sagittal skeletal classes were divided as Class I, Class II, and Class III as follows:

- Skeletal Class I: ANB angle between 0 and 4.
- Skeletal Class II: ANB angle above 4.
- Skeletal Class III: ANB angle less than 0 [19].

The sagittal relationship of the first molars was evaluated by examining intraoral photographs taken with a digital camera (Canon EOS 700D Digital SLR Camera, Canon Inc., Taiwan, Japan) before treatment. Therefore, dental classifications were divided as angle Class I, II, and III according to the molar relationship as follows:

- Angle Class I: neutral bite in which the mesiobuccal tubercle of the maxillary first molar fits into the buccal sulcus of the mandibular first molar.
- Angle Class II: a bite in which the mandibular first molar is more distal than in angle Class I.
- Angle Class III: a bite in which the mandibular first molar is more mesial than angle Class I [20].

**Figure 1:**
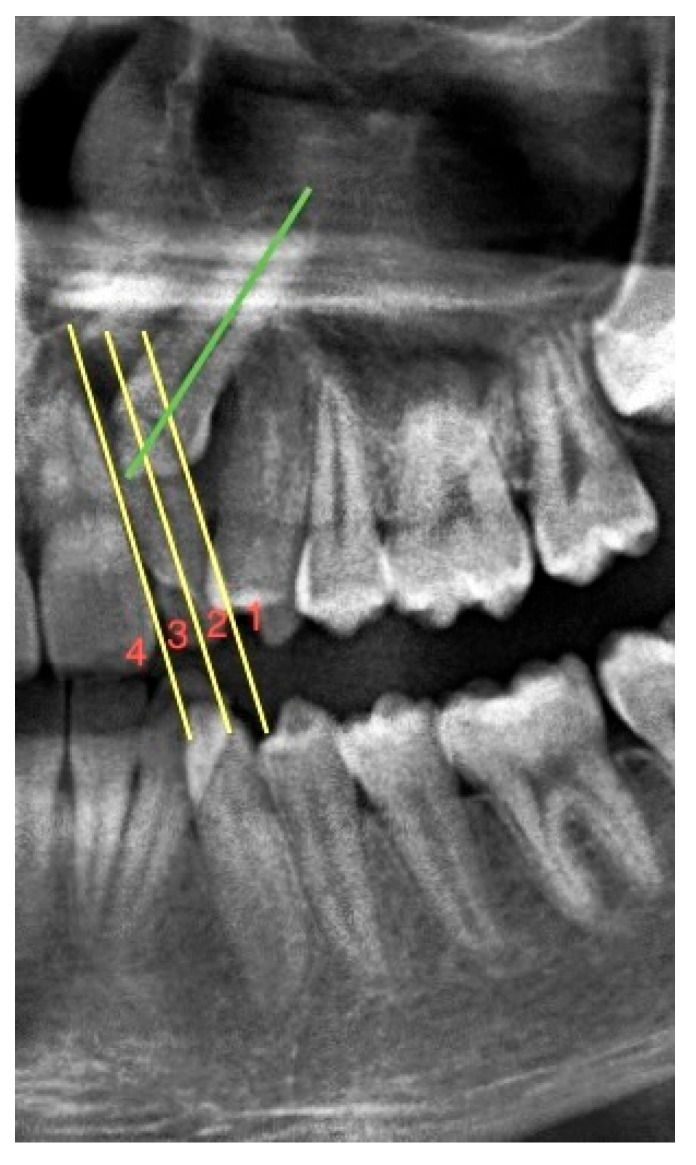
A section from the panoramic radiograph showing the sector classification of the impacted maxillary canine relative to the root of the lateral tooth. Green line: long axis of the impacted maxillary left canine. Yellow line: it divides the maxillary lateral incisor into 4 separate regions parallel to its long axis. (Sector classification of this impacted maxillary left canine is 3).

To determine the mesiodistal position of the impacted maxillary canines, the sector classification used by Lindauer et al. [21] concerning the permanent lateral incisors was used. Three vertical lines divide the lateral tooth and its surroundings into four sectors. The first of these lines passes through the center of the lateral tooth. The second line passes through the mesial root surface of the lateral tooth, and the third line passes through the distal root surface. In this way, impacted maxillary canines were classified according to these regions as sectors 1, 2, 3, and 4 from distal to mesial (see Figure 1).

**Figure 2:**
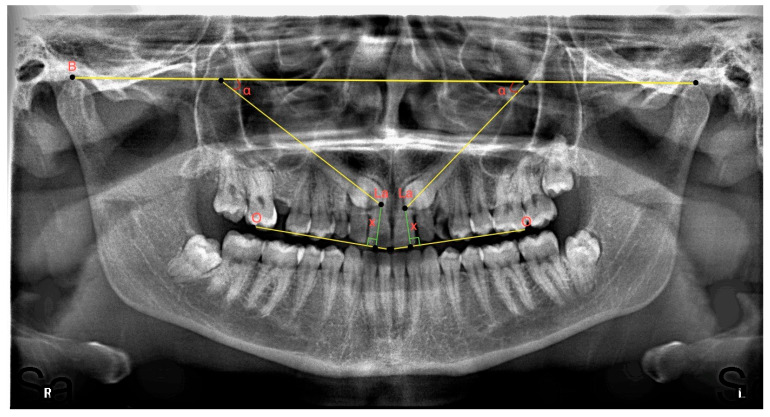
A panoramic radiograph showing the angle between the right and left impacted maxillary canines and the bicondylar plane, separately. **B**: Bicondylar plane, the junction of the uppermost and anterior points of the condyles; **La**: line passing through the long axis of the canines; **O**: occlusal plane; **x**: perpendicular distance of the cusp apex of the canine from the occlusal plane; and **α**: medial angle between the long axis of the canine and the bicondylar plane.

In determining the mesiodistal angular positions of impacted maxillary canines, the bicondylar plane drawn from the uppermost and anterior points of the right and left condyles was created as a fixed reference [22]. The medial angle between this reference line and the long axis of the canine was measured and recorded (see Figure 2).

On panoramic radiographs, the distance of the impacted maxillary canines to the occlusal plane was recorded by measuring the distance of the vertical line descending from the canine cusp apex to the occlusal plane [23] drawn between the maxillary central incisor and the mesial tubercle of the maxillary permanent first molar (see Figure 2).

### Statistical Analysis

The obtained data were analyzed statistically using the Statistical Package for the Social Sciences program (SPSS, version 26, IBM Corporation, New York, NY, USA). The normality distribution of the data was performed with the Kolmogorov–Smirnov test, the analysis of qualitative independent data using the Chi-square test, and the analysis of quantitative independent data using the Kruskal–Wallis and Mann–Whitney U tests. The reliability test between linear and angular measurements performed four weeks apart was evaluated with Cronbach’s α and two-way random effect intraclass correlation coefficients. The statistical significance level was accepted as *p* < 0.05.

## 3. Results

In repeated measurements performed four weeks later in 20 randomly selected samples, the intraclass correlation coefficients were 0.985 for canine distance and 0.993 for canine angle. These data revealed excellent intra-observer reliability in the measurements. According to the Kolmogorov–Smirnov test, the data were not normally distributed (*p* < 0.05).

The positions of the impacted maxillary canines were determined from cone-beam computed tomography (CBCT) as follows: while 62 (%65) of 96 impacted right maxillary canines were located palatally and 34 (%35) were located labially, 68 (%70) of 97 impacted left maxillary canines were located palatally and 29 (%30) were labial.

Impacted canine #13 was statistically significantly higher in females and lowest in males compared to impacted canine #23 and bilateral impacted canines. Bilateral impacted canines were significantly less in angle Class II patients than unilateral impacted canines, while it was significantly higher in skeletal Class III patients than in other skeletal classes (*p* < 0.05). No significant difference was found between impaction and age groups (*p* > 0.05) (Table 2).

Impacted canine angles were statistically significantly lower in females than males (*p* < 0.05). Impacted canine angles in the 13–16 age group were statistically significantly higher than those in the 17–20 and 21+ age groups (*p* < 0.05). Angle Class II was significantly lower than angle Class I and Class III, while there was no significant difference between the canine angles of angle Class I and Class III. No significant difference was found between skeletal classes and canine angles (*p* > 0.05). There were significant differences between sector classification and canine angles. Accordingly, the highest canine angle was significantly higher in sector 1, while the lowest was in sector 4. There was a statistically significant decrease in canine angles from sector 1 to sector 4 (*p* < 0.05). There was no significant difference between the impaction angles of teeth 13 and 23 (*p* > 0.05) (Table 3).

There were no statistically significant differences between gender, age groups, and skeletal classes regarding the occlusal plane distance of impacted canines (*p* > 0.05). The distance of the canines to the occlusal plane in angle Class II was significantly greater than in Class I, whereas there was no significant difference between the distance of the canines in angle Class I and Class III to the occlusal plane. The distance to the occlusal plane of the impacted canines in sector 4 was significantly higher than in sectors 1 and 2 (*p* < 0.05). No significant difference existed between the occlusal plane distances of impacted canines 13 and 23 (Table 4).

## 4. Discussion

In this study, while bilateral maxillary canine impaction was mostly seen in skeletal Class III, no significant difference was found between age groups. In addition, it was found that impacted maxillary canine angles were less in dental Class II, but there was no significant difference between skeletal classes. Again, with the lowest angle in sector 4, it was observed that the angle of the impacted maxillary canines from sectors 1 to 4 was significantly reduced due to more horizontal positioning. Thus, it was seen that the first null hypothesis of the study was partially rejected.

Describing various linear and angular measurements that can successfully predict impacted canines would be useful for the clinician to take timely measures. Therefore, the diagnostic methods of impacted canines can predict the difficulty of orthodontic treatment, duration, and possible treatment options. Diagnosis starts with clinical examination and continues with radiographic evaluations [24]. This study reveals the relationship between impacted maxillary canines with malocclusion during orthodontic diagnosis. In this context, while the distances of the impacted maxillary canines to the occlusal plane did not differ significantly between gender, age groups, and skeletal classes, it was found to be significantly higher in dental Class II and sector 4. This showed that the second null hypothesis of the study was also partially rejected.

The best option for determining the position of impacted canines is CBCT, which, although costly, provides a reliable three-dimensional evaluation of impacted canines and improves positional accuracy [24,25]. Panoramic and lateral cephalometric radiographs are usually taken from patients following orthodontic treatment and minimize radiation exposure. These radiographs provided sufficient data for the present study [7], and panoramic radiographs were used to evaluate impacted canines’ angular and linear position.

Arandi et al. [26] investigated the prevalence of impacted maxillary canines on panoramic radiographs of 1321 Palestinian individuals aged between 15 and 67 and found a statistically significant relationship between gender and canine impaction. Accordingly, females have a higher incidence of impacted canines than males. The same researchers also found that unilateral canine impaction (79%) was significantly higher than bilateral impaction (21%). In this present study of 151 Turkish individuals living in the Western Black Sea Region, it was found that maxillary impacted canines were more common in females than males, and unilateral impacted canines were more common than bilateral impacted canines.

Jameel et al. [27] evaluated the relationship between gender and impacted area in a study of 500 patients in Peshawar aged between 15 and 25 years who presented for orthodontic treatment. They reported that the left maxillary canine most commonly impacted males and females. They reported the most common left maxillary canine impaction in both males and females. Unlike the previous study, in this study, impaction of the right canine was found to be highest in females and lowest in males.

In a study conducted by Pop et al. [7] to determine the types of malocclusion associated with impacted canines, they found that impacted canines were most commonly associated with Class II malocclusion and sector 4 according to Lindauer’s sectoral classification, which is consistent with this present study. It is thought that the reason for this may be that as the relationship of the canine tooth with the lateral tooth increases, the possibility of becoming impacted increases.

In a study conducted by Ismail et al. [22] to determine the position of maxillary canines in children and to predict the risk of impacted maxillary canines, no statistically significant difference was found between the angles of impacted canines numbered 13 and 23 with the bicondylar plane. The present study found no significant difference between the impacted angles of canines numbered 13 and 23.

In the literature, in contrast to studies examining the incidence and prevalence of impacted maxillary canines and their relationship with malocclusion, there are also studies examining local complications caused by maxillary canines, such as lateral tooth resorption [28,29]. In one of these studies, Dağsuyu et al. [30] evaluated the localization, angulation, and resorption characteristics of maxillary impacted canines with CBCT and found no statistically significant difference between the distances of right and left impacted canines to the occlusal plane, similar to the findings in the present study. In addition, in the current study, which investigated the relationship of impacted maxillary canines with the adjacent lateral tooth without evaluating the resorption findings, the distance of the impacted maxillary canines from the occlusal plane in sector 4 was found to be significantly higher than those in sectors 1 and 2.

In a study conducted by Nieri et al. [12], a surgical-orthodontic approach was used to treat 168 patients with intraosseous impacted maxillary canines aged 12.8 to 52 years old. The study aimed to assess the diagnosis of impacted maxillary canines and identify the potential factors that could impact the final treatment outcome. Accordingly, they found that the distance of the tooth to the occlusal plane decreased with increasing age at the beginning of treatment. In contrast to the present study, there was no statistically significant difference between the age groups and the distance of the impacted tooth to the occlusal plane. It is thought that this may be due to the different age ranges of the patients who constituted the study material and the differences in age grouping.

Malik et al. [23] performed angular and linear measurements to determine the position of the impacted canine on the panoramic radiographs of the patients to help early diagnosis of palatally impacted canines and to determine the parameters of panoramic radiography. Their study found that as the sector class of the impacted canine increased, the distance to the occlusal plane increased, and the angle with the occlusal plane decreased, the teeth resulted in impacted canines. In this study, it was found that as the sector class of the impacted canine increased, the canine angle decreased significantly and the distance to the occlusal plane increased significantly, consistent with the study of Malik et al.

Comparison of existing studies in the literature is complicated and complex due to differences in sample size, grouping methods, clinical examination methods, and changes in radiographic techniques used for diagnosis [10]. Therefore, although the findings of this study provide important data for the literature, there are some limitations. In the etiology of impacted maxillary canines, local factors include tooth size incompatibilities, space limitations in the maxilla due to early loss of primary teeth, and contact with lateral teeth [31]. The limiting factors of this study were that the etiologic reasons for the maxillary canines to be impacted were not investigated, the lack of knowledge of the existing crowding/diastema/transversal discrepancy in the maxilla, and the angles and distances of the impacted maxillary canines were measured by two-dimensional X-rays without determining the exact three-dimensional position in the bone. In addition, panoramic radiographs are a two-dimensional dental X-ray examination that can obtain well-defined records of all teeth and adjacent structures and play an important role in the diagnosis and treatment planning of a wide variety of dental and maxillofacial diseases [32,33]. Furthermore, the panoramic radiographs used in this study were reported to be reliable for determining the position of impacted maxillary canines, especially in the middle and coronal regions [34].

The fact that there is a significant relationship between dental and skeletal malocclusions and impacted maxillary canines in the study necessitated radiographic examination and angular evaluation of permanent maxillary canines in the presence of skeletal Class III and dental Class II, even if the eruption time has not come. Early orthodontic interventions such as rapid and/or slow transversal expansion, tooth extraction, and incisor or molar/premolar uprighting can be effective in gaining space and guiding the eruption of maxillary canines [35]. In the future, the follow-up of both maxillary canine eruptions and growth and development may be more predictable with diagnostic tools that can simulate growth and development, taking into account the existing three-dimensional skeletal pattern and dental occlusion.

## 5. Conclusions

Within the limits of the study, which found a significant relationship between malocclusions and impacted maxillary canines, the following conclusions were reached:

✓ Maxillary canine impaction is approximately two times more common in females than in males.
✓ Unilateral impaction is more common than bilateral impaction.
✓ Bilateral maxillary canine impactions are more common in skeletal Class III patients than unilateral impactions.
✓ As the relationship of the impacted maxillary canine to the adjacent lateral tooth increases (going from sector 1 to 4), the angle decreases, the distance to the occlusal plane increases, the tooth transitions to a more horizontal impacted position, and the probability of the tooth remaining impacted increases.
✓ The canine angle is lower in females and dental Class II patients.
✓ Evaluation of skeletal and dental status with early orthodontic examination provides important prediction of impaction of maxillary canines.

## Figures and Tables

**Table 1:** Characteristics of the included patients.

	Females	Males	Total
Gender	101 (67%)	50 (33%)	151 (100%)
Age (mean ± SD)	17.06 ± 3.9	15.5 ± 2.6	16.56 ± 3.6
Right maxillary canine impacted patient	45 (30%)	9 (6%)	54 (36%)
Left maxillary canine impacted patient	31 (20%)	24 (16%)	55 (36%)
Bilateral canine impacted patient	25 (17%)	17 (11%)	42 (28%)
Total canine impacted patient	101 (67%)	50 (33%)	151 (100%)

%: percent, SD: standard deviation.

**Table 2:** Statistical analysis results of canine impaction according to gender, angle molar classification, age, and skeletal classification.

	Groups	Impacted Canine #13	Impacted Canine #23	Bilateral Impacted Canines #13, 23	Total	X^2^	df	*p*
Gender	Female Male	45 ^a^ 9 ^a^	31 ^b^ 24 ^b^	25 ^b^ 17 ^b^	101 50	10.373	2	0.006 *
Angle molar classification	Class I Class II Class III	18 ^a^ 27 ^a,b^ 9 ^a^	17 ^a^ 34 ^b^ 4 ^a^	22 ^a^ 12 ^a^ 8 ^a^	57 73 21	11.670	4	0.02 *
Age	13–16 years 17–20 years Over 21 years	29 ^a^ 16 ^a^ 9 ^a^	33 ^a^ 13 ^a^ 9 ^a^	32 ^a^ 5 ^a^ 5 ^a^	94 34 23	5.758	4	0.218
Skeletal classification	Class I Class II Class III	36 ^a^ 11 ^a^ 7 ^a^	37 ^a^ 13 ^a^ 5 ^a^	23 ^a^ 5 ^a^ 14 ^b^	96 29 26	11.491	4	0.02 *
	Total	54	55	42	151			

X^2^: Chi-square value; df: degree of freedom; *p*: significance level; *: *p* < 0.05; ^a,b^: There is a statistically significant difference between impacted groups with different top index letters in the same row; 13: maxillary right canine; and 23: maxillary left canine.

**Table 3:** Statistical analysis results according to the angle of the impacted canines between the groups.

	Groups	N	Mean ± SD	Canine Angle (Mean Ranks)	*p*
Gender	Female ^1^	126	61.78 ± 19.17	86.29 ^2^	<0.001 * ^K^
	Male ^2^	67	71.01 ± 16.07	117.14 ^1^	
Dual differences	*p*			0.000 * ^m^	
Age	13–16 years ^1^ 17–20 years ^2^ Over 21 years ^3^	123 43 27	68.88 ± 17.98 59.38 ± 17.40 56.18 ± 18.84	108.96 ^2,3^ 79.03 ^1^ 71.15 ^1^	<0.001 * ^K^
Dual differences	*p*			0.002 * ^m^	
Angle molar classification	Class I ^1^ Class II ^2^ Class III ^3^	80 85 28	68.58 ± 16.99 59.63 ± 19.31 70.98 ± 17.31	106.33 ^2^ 81.68 ^1,3^ 116.86 ^2^	0.002 * ^K^
Dual differences	*p*			0.005 * ^m^	
Skeletal classification	Class I ^1^ Class II ^2^ Class III ^3^	120 34 39	63.37 ± 18.93 64.68 ± 18.54 70.23 ± 17.26	92.40 94.15 113.63	0.113 ^K^
Dual differences	*p*			0.127 ^m^	
Sector classification	Sector 1 ^1^ Sector 2 ^2^ Sector 3 ^3^ Sector 4 ^4^	64 38 15 76	78.74 ± 14.61 69.90 ± 13.05 61.96 ± 9.56 51.54 ± 15.82	142.87 ^2,3,4^ 109.49 ^1,3,4^ 79.87 ^1,2,4^ 55.51 ^1,2,3^	<0.001 * ^K^
Dual differences	*p*			0.000 * ^m^	
Impaction	Impacted canine #13 ^1^	96	64.61 ± 18.37	95.40	0.691 ^K^
	Impacted canine #23 ^2^	97	65.36 ± 18.98	98.59	
Dual differences	*p*			0.691 ^m^	

^K^: Kruskal–Wallis test; ^m^: Mann–Whitney U test; N: number of impacted maxillary canines; Mean ± SD: mean ± standard deviation; ^1,2,3,4^: there is a statistically significant difference between groups with different top index numbers in the same row; *p*: significance level; and *: *p* < 0.05.

**Table 4:** Statistical analysis results according to the occlusal plane distance of impacted canines between the groups.

	Groups	N	Mean ± SD	Canine Distance (Mean Ranks)	*p*
Gender	Female ^1^	126	9.89 ± 3.75	97.38	0.897 ^K^
	Male ^2^	67	9.89 ± 3.71	96.28	
Dual differences	*p*			0.897 ^m^	
Age	13–16 years ^1^ 17–20 years ^2^ Over 21 years ^3^	123 43 27	9.69 ± 3.87 10.40 ± 3.43 10.01 ± 3.51	93.04 106.08 100.57	0.393 ^K^
Dual differences	*p*			0.534 ^m^	
Angle molar classification	Class I ^1^ Class II ^2^ Class III ^3^	80 85 28	9.21 ± 3.48 10.76 ± 3.89 9.25 ± 3.42	86.89 ^2^ 109.11 ^1^ 89.13	0.028 * ^K^
Dual differences	*p*			0.013 * ^m^	
Skeletal classification	Class I ^1^ Class II ^2^ Class III ^3^	120 34 39	9.90 ± 3.94 9.75 ± 3.82 10.06 ± 2.95	95.67 95.76 102.18	0.810 ^K^
Dual differences	*p*			0.527 ^m^	
Sector classification	Sector 1 ^1^ Sector 2 ^2^ Sector 3 ^3^ Sector 4 ^4^	64 38 15 76	8.74 ± 4.14 9.19 ± 2.72 9.65 ± 2.81 11.26 ± 3.56	76.40 ^4^ 86.97 ^4^ 97.60 119.24 ^1,2^	<0.001 * ^K^
Dual differences	*p*			0.001 * ^m^	
Impaction	Impacted canine #13 ^1^	96	9.43 ± 3.56	91.20	0.151 ^K^
	Impacted canine #23 ^2^	97	10.35 ± 3.84	102.74	
Dual differences	*p*			0.151 ^m^	

^K^: Kruskal–Wallis test; ^m^: Mann–Whitney U test; N: number of impacted maxillary canines; Mean ± SD: mean ± standard deviation; ^1,2,3,4^: there is a statistically significant difference between groups with different top index numbers in the same row; *p*: significance level; and *: *p* < 0.05.

## Data Availability

All data supporting the results of this study are included within the article. The data are currently not publicly available as they will be used in another study still in progress.
